# Complexity Changes in Brain Activity in Healthy Ageing: A Permutation Lempel-Ziv Complexity Study of Magnetoencephalograms

**DOI:** 10.3390/e20070506

**Published:** 2018-07-03

**Authors:** Elizabeth Shumbayawonda, Pinar Deniz Tosun, Alberto Fernández, Michael Pycraft Hughes, Daniel Abásolo

**Affiliations:** 1 Centre for Biomedical Engineering, Department of Mechanical Engineering Sciences, Faculty of Engineering and Physical Sciences, University of Surrey, Guildford GU2 7XH, UK; 2 Laboratorio UPM-UCM de Neurociencia Cognitiva y Computacional, Universidad Complutense de Madrid-Universidad Politécnica de Madrid. Departamento de Medicina Legal, Psiquiatría y Patología, Universidad Complutense de Madrid, Madrid 28040, Spain

**Keywords:** magnetoencephalography, permutation Lempel-Ziv complexity, life span, healthy ageing

## Abstract

Maturation and ageing, which can be characterised by the dynamic changes in brain morphology, can have an impact on the physiology of the brain. As such, it is possible that these changes can have an impact on the magnetic activity of the brain recorded using magnetoencephalography. In this study changes in the resting state brain (magnetic) activity due to healthy ageing were investigated by estimating the complexity of magnetoencephalogram (MEG) signals. The main aim of this study was to identify if the complexity of background MEG signals changed significantly across the human lifespan for both males and females. A sample of 177 healthy participants (79 males and 98 females aged between 21 and 80 and grouped into 3 categories i.e., early-, mid- and late-adulthood) was used in this investigation. This investigation also extended to evaluating if complexity values remained relatively stable during the 5 min recording. Complexity was estimated using permutation Lempel-Ziv complexity, a recently introduced complexity metric, with a motif length of 5 and a lag of 1. Effects of age and gender were investigated in the MEG channels over 5 brain regions, i.e., anterior, central, left lateral, posterior, and, right lateral, with highest complexity values observed in the signals recorded by the channels over the anterior and central regions of the brain. Results showed that while changes due to age had a significant effect on the complexity of the MEG signals recorded over 5 brain regions, gender did not have a significant effect on complexity values in all age groups investigated. Moreover, although some changes in complexity were observed between the different minutes of recording, due to the small magnitude of the changes it was concluded that practical significance might outweigh statistical significance in this instance. The results from this study can contribute to form a fingerprint of the characteristics of healthy ageing in MEGs that could be useful when investigating changes to the resting state activity due to pathology.

## 1. Introduction

It is a well-known fact that the delicate, anatomically intricate, and structurally compact nature of the brain combined with the different tissues surrounding it, such as the cerebral-spinal fluid, skull and skin, make this organ a very complex one to study both invasively and non-invasively. As the brain grows until the end of maturation (usually until the third decade), it undergoes various anatomical and physiological changes, in axonal volume, myelination, synaptic density, volume, and, neurotransmitter levels. Numerous studies on the maturation and ageing of the brain have highlighted this [1,2,3,4].

The use of non-invasive technologies to analyse brain activity is an essential part of research in neuroscience and biomedical engineering. Non-invasive techniques such as: functional magnetic resonance imaging (fMRI) [5], computed tomography perfusion (CTP) [6], positron emission tomography (PET) [7], electroencephalography (EEG) [8], magnetoencephalography [4], and near infrared spectroscopy (NIRS) [9], have been used to image/record brain/brain activity to obtain a more detailed understanding of its function. Moreover, the use of these techniques to investigate the brain has led to the generation of essential information about the effects of age [7], gender [3], pathology [6], and disease progression [6] on the brain. However, although all these brain imaging techniques are valuable in their own right, the use of magnetoencephalography as an imaging technique has increased over the years, in spite of its high cost. Magnetoencephalogram (MEG) signals provide a reasonable measure of the resultant activity of neurons in the brain (lying in the vicinity of the recording sensor [10]), which play an important role in the maintenance of the metastable state of the resting brain [11]. MEG analysis has good spatial and temporal resolution and has proved to be a robust method both in research and clinical settings. Nevertheless, similar to EEG, MEG analysis at the sensor level is affected by volume conduction, although to a much smaller degree [12]. MEG data have been used to: identify epileptic zones [13], investigate brain function, evaluate neurofeedback [14], determine the effects of pathology, investigate effect of cognitive decline [15], and, to determine the effects of ageing on the brain [16].

Once the brain recordings are obtained, a wide range of linear and non-linear and processing techniques, such as causality [17], entropy [16], synchronisation [18], correlation [19], and complexity [4], can be applied to them. Complexity is a concept stemming from non-linear analysis methods that could be applied to evaluate changes in brain activity recorded in MEG signals (see [20] for a detailed description of complexity). The complexity of the MEG signal can act as a robust indicator of the variances in cortical neuronal interaction as changes in complexity may, direct/indirectly, be caused by changes in the cortical functional organization of the brain [21]. Therefore, it is possible that the complexity changes of the MEG signal may be coarsely related to the changes in the regularity of the interactions occurring within the neuronal network [21].

Among the different algorithms to estimate complexity, it is worth noting that those based on symbolic dynamic methods are computationally efficient and have been successfully used to analyse the activity of the brain, with results showing changes in the brain activity due to age [2], gender [4], and, pathology [21]. For instance, permutation entropy (PE) [22], symbolic transfer entropy [23], Lempel-Ziv complexity (LZC) [4,24], and permutation Lempel-Ziv complexity (PLZC) [25] are all symbolic dynamic methods which have been used to investigate brain activity. In this investigation, PLZC was selected to estimate the complexity of the MEG time series. PLZC was introduced by Bai et al. [25] and is a complexity estimation method that combines permutation entropy (PE) and LZC to quantify the dynamic changes in signal complexity. The use of PLZC to estimate complexity is advantageous as it combines the benefits of using the classic LZC algorithm, which reflects the magnitude of the signal points and is frequency sensitive [24], with the benefits of using PE, which takes into account the temporal order/mutual relationship of the signal points [25]. Therefore, PLZC is a useful technique to use in characterising changes in the brain dynamics, as these changes are usually accompanied by changes in MEG frequency [26]. An additional advantage to the use of PLZC is that the method does not require stationarity on the time series [27]. However, as PLZC uses permutations that are derived from the mutual relationship between a data point and its neighbours, this makes the method sensitive to data sampling frequency. Nevertheless, as PLZC can give reliable results using relatively short signal segments, it is possible that, in the face of down-sampling, the use of relatively long epochs will give reliable results, as long as the sampling frequency of the signal is adequate and follows Nyquist criterion.

It was our hypothesis that PLZC would identify any differences in complexity due to age and gender in MEGs recorded during resting state. The study of the resting state is very relevant as it is possible that subtle changes to the background brain network, which would otherwise be hidden during tasks, can be detected. For instance, studies such as those done by Lopez et al. [28] using MEG resting state data, as well as by [29] using resting state fMRI (rs-fMRI) have successfully shown the importance of using resting state data when investigating the functioning of the brain. Hence with the overall aim of identifying the changes in complexity (measured by PLZC) of the background MEG signals across the human lifespan, the objectives of this study included: the choice of optimal input parameters for the computation of PLZC, the investigation to identify the stability of complexity values during relatively short recordings (so as to determine if the brain activity stayed relatively constant when in resting state), and the investigation of the effects of age and gender on the complexity of MEG signals.

The structure of this paper is as follows: Section 2 contains a description of the MEG data used as well as brief descriptions of the PLZC algorithm, and the results from the study are presented and described in Section 3. Section 4 has a detailed discussion of the results and their relevance, and the conclusions of the study are presented in Section 5.

## 2. Materials and Methods 

A database of MEG recordings from 177 healthy subjects (79 males/98 females) with ages ranging between 21 and 80, and with no significant differences in terms of age between males (mean ± std 48.36 ± 19.93) and females (51 ± 18.26) was used in this study. Table 1 shows how subjects were classified into the difference groups used, as well as the number of subjects in each age group. In addition, Table 1 also highlights how some of the age groups can be separated into different categories based on different stages of life and brain growth described by [30,31,32].

During the collection of the MEG recordings of the database used in this study, subject inclusion criteria included the absence of: neurological-psychiatric-developmental disorders, drug disorders, drug consumption, evidence of substance abuse, and, head trauma. Moreover, controls were defined in this manner, with no other characteristics such as high education levels, intelligence or IQ used as subject inclusion criteria.

MEG data were acquired in a magnetically shielded room, at the Centro de Magnetoencefalografía Dr. Pérez-Modrego (Madrid, Spain), using a 148 channel (MAGNES 2500WH, 4D Neuroimaging) neuroimaging magnetometer with a hardware band-pass filter and sampling frequency of 678.17 Hz. The 5 min MEG recordings were then down-sampled by a factor of 4, to 169.549 Hz, after which the data were then filtered using a 560 order band-pass filter (digital Hamming window finite impulse response filter applied using the filtfilt function to avoid phase shift (MATLAB version 2016a)) with corner frequencies at 1.5 Hz and 40 Hz [33]. Data were down-sampled, following Nyquist criteria, to enable ease of storage and faster analysis while maintaining all necessary information, while bandpass filtering was used to eliminate noise and unwanted signals from the data (such as electrocardiograms (ECG), electrooculogram (EOG), eye-blinks, and electromyograms (EMG)). Although other methods such as independent component analysis (ICA) could have been used to remove noise artefacts, these methods were not used in this study due to the loss of data associated with such techniques [34,35].

### 2.1. Permutation Lempel-Ziv Complexity

Permutation Lempel-Ziv Complexity (PLZC) is based on the Lempel-Ziv Complexity (LZC) algorithm, that uses permutations (motifs) of a chosen length *m* to estimate the complexity of a signal instead of applying a coarse graining procedure to the signal [25]. As explained extensively by Bai, et al. [25] different combinations of motif length (*m*) and time delay (*τ*) can be used to estimate the complexity of a signal, with their selection being dependent on the nature and length of the signal being investigated. Smaller values of *m* are more suitable (when used in combination with low values of *τ* such as *τ* = 1) to investigate short signals with fast changing dynamics, while larger values of *m* are suitable for longer signals with slower changing dynamics. Thus, selection of the motif length and time delay is very dependent on the signal acquisition parameters as well as the signal dynamics.

To estimate complexity using the PLZC algorithm the number of possible motifs, given by m! in the permutation symbolising procedure must be less than the time series length α such that if LZC is given by [36]:

(1) $$\;C\left(n\right)=c\left(n\right)\left[log_{\alpha }\left\{c\left(n\right)\right\}+1\right]$$

Then PLZC can be defined as a normalised C(n) as follows, where n represents the total length of the time series and m!≤α:

(2) $$\;PLZC=\frac{c\left(n\right)\left[log_{m!}\left\{c\left(n\right)\right\}+1\right]}{n}$$

Furthermore, in the event of very long signals with large *n* (usually >9!), PLZC can be simplified and defined as:

(3)  PLZC=c(n)[logm!n]n

**Figure 1:**
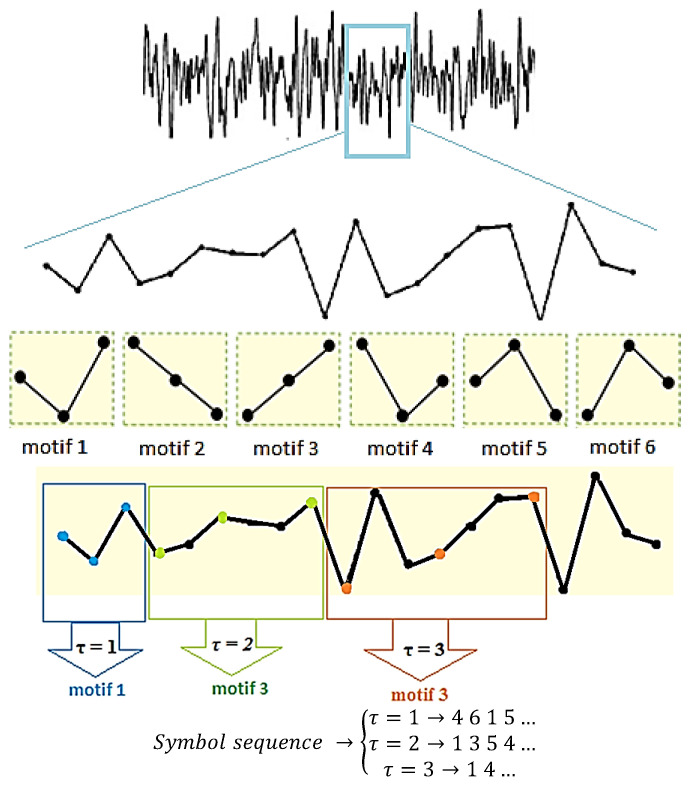
Illustration of the effects of using different input parameters during the symbolisation process of the PLZC algorithm on a short section of a time series. This example uses a motif length of 3 in combination with different time delay values. With *m* = 3, there are 6 (3!) possible motifs that can be used to estimate the complexity of the time series [25]. The symbol sequence shows the different motif combinations that can be obtained when using different values of *τ.*

To highlight further the dependence of the motif structures on the input parameters *m* and *τ*, Figure 1 shows a graphical example using a motif length of 3. Additionally, the effects of using different values of *τ* are also highlighted in this illustration that was adapted from Bai et al. [25].

The following approach was used to investigate the aims of this study. Firstly, investigations to evaluate the stability of PLZC values during recording were performed. To do this, 5 min of MEG data were divided into five 1 min long consecutive epochs containing >10,000 data points each. Complexity (PLZC) was then calculated for each minute for each subject before grouping, and all subjects with outlier complexity values were removed from the sample. As recommended by Bai et al. [25], different values of *m*, between 3 and 9, were investigated so as to identify the motif length to use in complexity estimation. Furthermore, to assist with the selection of an adequate value of *m*, colour maps were also plotted to act as a visual qualitative aid. As the data being used had already been down-sampled prior to applying PLZC, and the effect of changing the values of *τ* is similar to that of down-sampling, the value of *τ* was set to 1 for all calculations made in this study. It must be noted that the MEG sensors were grouped into regions, similar to work done by Shumbayawonda et al. [16] and Fernandez et al. [4], and shown in Figure 1. However, as this is a sensor space study, references made in the text to brain regions refer to the MEG data recorded by the channels over the respective brain regions.

After these investigations, age and gender effects (as well as their interaction effects) were then analysed using the PLZC values from the 5 min of recording (averaged from the 1 min epochs). Firstly, the PLZC values for each channel were plotted on a colour map so as to enable visualisation of the spread of the PLZC values across the brain. Channels were then divided into brain regions laying over the surface of the brain, after which PLZC values for each region were then calculated, and plotted, to enhance understanding of the differences between the regional PLZC scores of males and females, as well as the differences due to age. Further analysis were performed to explore the differences between gender PLZC values, with statistical analysis to determine the interaction effects of age and gender on the PLZC values in each brain region also performed.

### 2.2. Statistical Analysis

All statistical analyses were performed using IBM statistical package for the social sciences (SPSS) statistical data editor version 24, and probabilities *p* < 0.05 were considered as significant unless otherwise stated. Investigations to determine the similarities between the PLZC values obtained using different values of *m* (which would ultimately result in the selection of a motif length for use in the analysis) were performed using Spearman’s RHO index. PLZC results followed a normal distribution, and statistical analysis to determine the significance of complexity changes in each brain region, due to age and gender, were performed using 2-way analysis of variance (ANOVA). For the gender analysis, pairwise comparisons were corrected for using the least significant difference (LSD) adjustment for multiple comparisons, while in the age analysis, Bonferroni correction were used in the post hoc comparisons. A 2-way ANOVA was used to investigate the interactive effects of age and gender on PLZC values, while a 3-way ANOVA was used to investigate the interactive effect of age and gender in each brain region. Finally, a 1-way repeated measures ANOVA was used to determine the significance of the changes in complexity values during each minute of recording.

## 3. Results

### 3.1. Selection of Motif Length

The first part of this investigation focused on the identification of the motif length to use in the PLZC analysis. PLZC values were calculated for each subject for all 148 channels with *m* values of 3, 5 and 7. Statistical analysis to determine correlations between data obtained using the different motif lengths was used to narrow the options to enable easier selection of input parameters. Results from the analysis using Spearman’s RHO index are shown in Table 2 below. Each comparison was made separately between males and females in each age group, and results show that the PLZC values obtained using *m* = 5 and *m* = 7 were strongly correlated.

**Figure 2:**
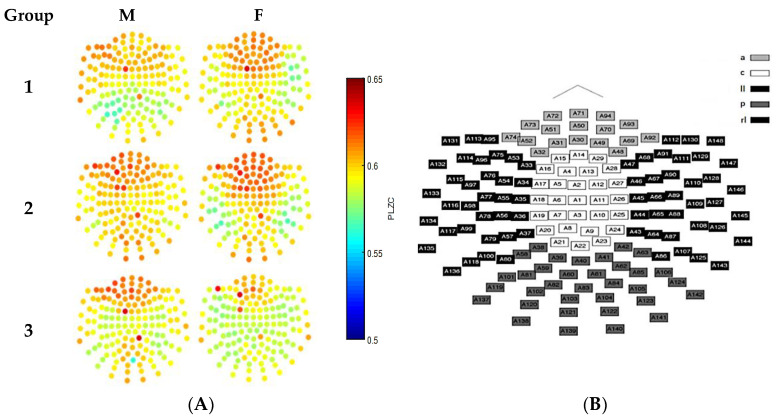
(**A**) Colour maps showing the average complexity results from the 5 min MEG recording using PLZC analysis for each SQUID channel. Results are shown for males (**left**) and female (**right**) for the 3 age groups using in this study; (**B**) Sensor-space representation of the layout of the location of the 148 SQUID channels in the neuromagnetometer used to record MEG signals. The five highlighted regions represent the sensor groupings used to define the different parts of the brain i.e., anterior (a), central (c), left lateral (ll), right lateral (rl) and posterior (*p*). These regions were also used for the statistical analyses [4].

In addition to the use of statistical analysis, qualitative analyses were also performed where the PLZC values were visualized using colour maps. Similarly, colour maps from results using *m* = 5 and *m* = 7 were very similar to each other when compared to those using *m* = 3. Thus, although qualitative analyses may not be used to determine statistical significance, they are an invaluable tool that can be used to understand and interpret data. Therefore, as a result, due to the large differences between the number of permutations used between *m* = 5 (5! = 120) and *m* = 7 (7! = 5040), and the high correlations between the results, a motif length of 5 was selected for use in this study as this would result in faster computation. Figure 2 is an illustration of the qualitative results obtained using *m* = 5. As can be seen in the figure, the colours represent the PLZC values for each channel, with lower PLZC values (represented by colder colours) implying less complexity in the data, and higher PLZC values (represented by warmer colours) implying higher complexity in the time series data.

### 3.2. Analysis of Short Term Variability of Complexity Values

Complexity changes between the five 1 min epochs were also investigated in this study. Comparisons between the complexity values of each channel in each epoch were performed for each age group and gender using 1-way repeated measures ANOVA with Bonferroni corrections (significance at *p* < 0.05/4) for multiple comparisons. For females, results showed that:

1. In group 1, there were no significant differences (*p* > 0.0125) between the complexity values of minutes 3 to 5, however minutes 1 and 2 contained complexity values that were significantly different to the other minutes of recording (*p* < 0.0125),
2. In group 2, only minute 1 had significantly different complexity values (*p* < 0.0125) when compared to the complexity values of the other 4 min of recording,
3. In group 3, minute 5 had significantly different PLZC values (*p* < 0.0125) when compared to other minutes, however, there were no significant differences between complexity values between minutes 1 and 4.

When this analysis was repeated for males, results showed that in:

1. Group 1, only minutes 1 and 2 had similar complexity values (*p* > 0.0125), while minutes 3 to 5 had significantly different values (*p* < 0.0125),
2. Group 2, although minutes 1, 4 and 5 did not have significantly different values (*p* > 0.0125), minutes 2, 3 and 4 also did not have any significantly different values (*p* > 0.0125), thus, no clear and obvious trends were found, as all minutes were observed to have varying PLZC values,
3. Group 3, similar to the observation made for females, minute 5 had complexity values that were significantly different to all other minutes of recording.

**Figure 3:**
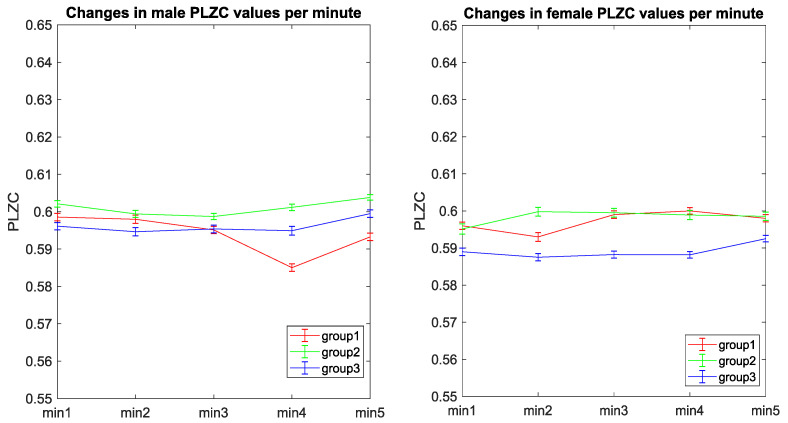
Changes in male (**left**) and female (**right**) average complexity (PLZC) values per minute during the 5 min MEG recordings. Each line represents the PLZC values from an age group, i.e., group 1 (red), group 2 (green), and group 3 (blue). Error bars representing the standard deviation of the mean are also shown in these trends.

Figure 3 shows an illustration of the changes in mean complexity values for males and females in the 3 age groups investigated. The plots shown in Figure 3 shows the mean values obtained in each epoch 1 min epoch. Evidently from the plots in Figure 3 show stability of complexity (PLZC) values during recording as there are very minimal difference (0.02) between the highest and lowest PLZC values across all three age groups (with even less variation within each age group).

**Figure 4:**
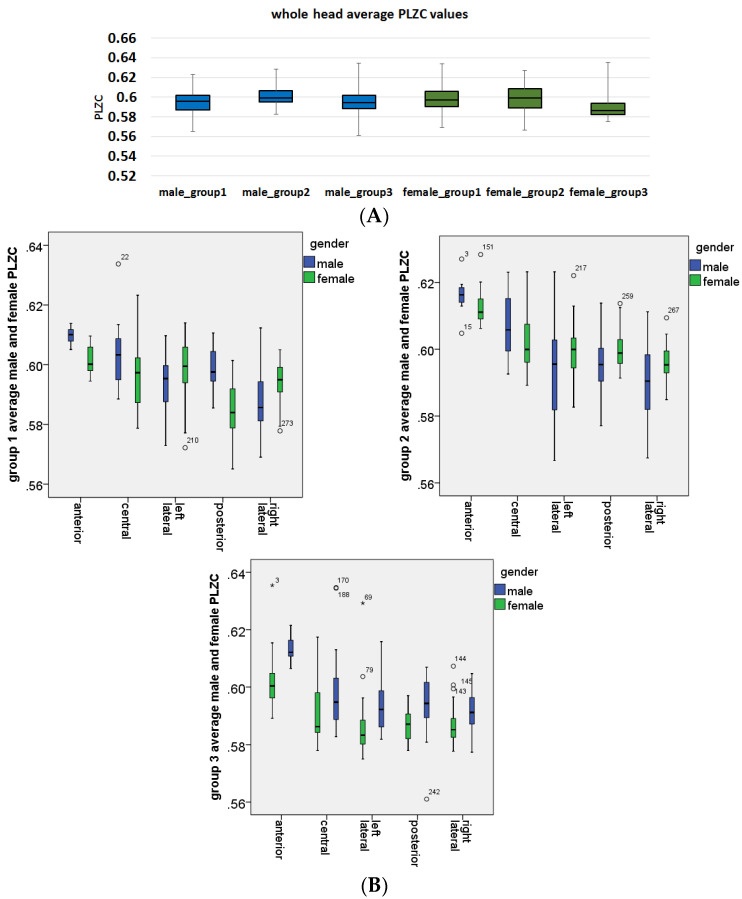

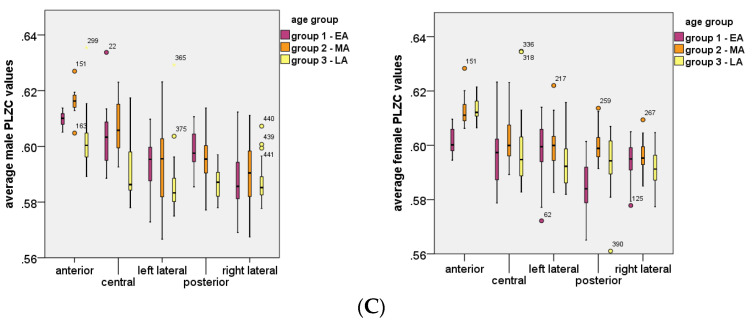
Illustration of the (**A**) average whole head complexity for males (blue) and females (green) for each age group; (**B**) Average complexity values for each brain region for each age group (group 1, group 2 and group 3). The plots highlight the differences in average PLZC values for both males and females in each brain region; (**C**) Average PLZC values for each age group shown separately for males (**left**) and females (**right**). The plots highlight the differences in average PLZC values with respect to age and are categorised by age group i.e., early adulthood (EA: group 1, purple), mid adulthood (MA: group 2, orange), and late adulthood (LA: group 3, yellow). Outlier channels are also highlighted in the box-and-whisker plots (male—blue, female—green) with circles indication mild outliers and stars representing extreme outliers.

Once a motif length was selected, the average whole head complexity values for each age group and gender were plotted to identify overall differences between complexity values, as shown in Figure 4A. It was found that although the complexity values overlapped, the mean values showed that subjects in mid-adulthood (group 2) had the highest PLZC values. Thus, more detailed investigation of the variation of complexity values for each gender group and in each brain region were performed, with results shown in Figure 4B. Evidently, this trend became clearer, and not only showed that subjects in the mid adult group had highest mean PLZC values, but also showed that channels over the anterior region had the highest complexity values. Gender differences were also investigated in the exploration stage, and it was found that males and females generally had similar values in each brain region. It was also found that in group 3 (late-adulthood), male mean complexity values were higher than female values, as shown in Figure 4C.

Once the investigations were complete, statistical analysis to determine the effects of age, gender, and the age and gender interactions in PLZC values ensued. Analysis to evaluate the short term variability of complexity values during resting state recordings was also performed.

### 3.3. Analysis of Gender Effects

**Figure 5:**
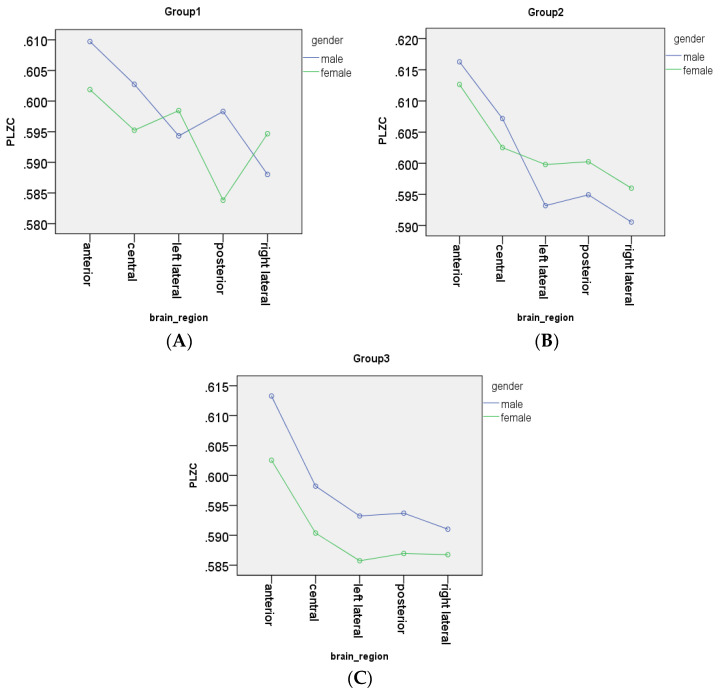
Illustration to highlight the differences between male and female average PLZC values over channels in each region for each age group. Male trends are shown in blue, while female trends are shown in green. Plots are classified by group, i.e., (**A**) group 1 (early-adulthood); (**B**) group 2 (mid-adulthood); (**C**) group 3 (late-adulthood).

A 2-way ANOVA test was used to determine the effects of gender on each brain region for all 3 age groups. For group 1 (21–40 years), it was found that gender had on overall significant difference between subjects effect (*p* = 0.00038). However, further analysis into the significance of gender effects per brain region showed that gender had a significant effect in all regions apart from the left lateral with significance values of p_a_ = 0.0098, p_c_ = 0.0013, p_ll_ = 0.054, p_p_ < 0.0001, and p_rl_ = 0.002 suggesting higher complexity values for males. Unlike group 1, gender did not have an overall significant between subjects in group 2 (41–60 years; *p* = 0.092). Additionally, regional investigations showed that of all 5 brain regions, only the anterior region did not show significant gender differences (suggesting differences in complexity) after post-hoc corrections (p_a_ = 0.235, p_c_ = 0.047, p_ll_ = 0.002, p_p_ = 0.014, and p_rl_ = 0.012). Lastly, for group 3 (61–80 years), gender showed a very strong overall significant effect with *p* < 0.0001, with regional analysis showing strong significant differences in all brain regions (p_a_ = 0.00039, p_c_ = 0.00069, p_ll_ = 0.00046, p_p_ = 0.0016, and p_rl_ = 0.045). Results from these gender analyses are shown in Figure 5.

### 3.4. Analysis of Age Effects

Figure 4C highlights the effects of age on PLZC values. As is evident from the plots the progression of PLZC values seems to follow an inverted U trajectory (similar to that observed by [4]) whereby PLZC increase from EA to MA and then subsequently decreases from MA to LA. This trend is was observed for both males and females, and when statistical analyses were performed, using 2-way ANOVA to determine effects of age in each brain region on PLZC values with Bonferroni corrections in post hoc analyses, age was found to have a significant effect with *p* < 0.0001 in all brain regions except for in the left lateral regions for males (*p* > 0.017).

### 3.5. Analysis of Age and Gender Interaction Effects

Further investigations of the individual effects of age and gender, using a 2-way ANOVA were performed to determine the between subjects interaction effect of age and gender on whole head PLZC values. This analysis was performed between males and females in each age group, and results showed that age and gender has significant interaction effects in group 1 (*p* = 0.000345) and group 3 (*p* < 0.0001), but not in group 2 (*p* = 0.0876). Thus, to understand these results further a 3-way ANOVA was performed to analyse the effects of age and gender in each brain region. Results from this analysis showed the following:

- In the anterior region, group 2 (*p* = 0.229) did not have a significant age and gender interaction effect on PLZC values., However, in group 1 (*p* = 0.0095) and group 3 (*p* = 0.000398) there was a significant interaction effect, which showed that PLZC values in this brain region (for both age groups) were influenced by gender, with complexity values being higher in males than in females.
- In the central region, group 1 (*p* = 0.0012), group 2 (*p* = 0.0441) and group 3 (*p* = 0.00717) all had significant interaction effects, which showed that males had higher PLZC values than females, and therefore gender influenced complexity values as a function of age for all three groups.
- In the left lateral region, group 2 (*p* = 0.002; females PLZC values were greater than males) and group 3 (*p* = 0.00047; male PLZC values were greater than females) had a significant interaction effect on PLZC values, while group 1 (*p* = 0.053) did not.
- In the posterior region, all groups had a significant interaction effect on PLZC values, with p_group1_ < 0.0001, p_group2_ = 0.0129, and p_group3_ = 0.00163. Thus, gender has a significant influence on PLZC values as a function of age, with males having higher complexity in group 1 and 3, and females having higher complexity in group 2.
- In the right lateral region, all groups had a significant age and gender interaction effect on PLZC values, with p_group1_ = 0.00189, p_group2_ = 0.0109, and p_group3_ = 0.0463. Gender has a significant effect as a function of age in all three groups, with females having higher complexity in group 1 and 2, while males had higher complexity values in group 2.

## 4. Discussion

In this study, PLZC (a new method based on the combination of concepts from PE and LZC) was used to analyse MEG signals obtained during resting state, across adulthood. The effects of age and gender were investigated, as well as their interactive effects, in each brain region. Both quantitative and qualitative analyses were used to interpret the results that were determined from this study. However, before these analyses were performed, input parameters of motif length (*m*) and time delay (*τ*) were carefully selected. As suggested by Bai et al. [25] and Bandt and Pompe [22], it is important to select the correct motif length for the time series being analysed, as this parameter has an effect on the permutation length, number of permutations used, and, ultimately, the complexity calculated and the interpretation of the results [37]. Although several studies have shown that the use of time delay of 1 can result in the extraction of useful data from the time series recording of brain activity [16,21,22,25,38,39], we chose *τ* = 1 to avoid any effects related to down-sampling.

Resting state is an intermediate stage between full mental engagement, as seen when performing a task, and full mental disengagement, as seen with sleep [26,40]. Thus, although this is a transition stage as suggested by Rubinov et al. [41], Deco et al. [42], and Stam et al. [11], it is not an entirely “constant” steady state as the brain is always modifying itself to ensure that it operates efficiently and economically [43,44]. Thus, with this in mind, in this study the stability of the complexity values throughout MEG recordings was also investigated.

### 4.1. Short Term Variability of Complexity Values

The short term variability of PLZC values was investigated to observe if these values remain relatively stable during the recording time. Moreover, as the resting state is usually defined by the parameters imposed in the subjects, and not usually the ‘regularity’ of their brain activity, this analysis was necessary in this study as it enabled a more accurate description of the resting state. Results showed that for females, subjects in group 1 (EA) had stable complexity values from minute 3 onwards, while those in group 2 (MA) had stable values from minute 2 onwards, and those in group 3 had stable complexity values between minutes 1 and 4. However, the analyses showed that for males, subjects in group 1 had stable complexity values in the first two minutes of recording, while those in group 2 did not have stable complexity values, and those in group 3 had stable values in the first four minutes of recording. However, it is interesting to note that apart from group 3, females had longer periods of “complexity stability” than males.

It is a well-known fact that to maintain homeostasis, the body operates between close levels of normality, this is evident with normothermia (lying between 36.5° and 37.5°) [45], resting heart rate (lying between 60–100 beats per minute) [46], and resting blood pressure (lying between 120/80 mm Hg—140/90 mm HG) [47]. In this study, complexity changes in short term recordings were evaluated and we found that complexity values fluctuated slightly between 0.585 and 0.605 (range of 0.02) around a mean value for healthy adults, as shown in Figure 5. Thus, there is a possibility that the electromagnetic activity making up resting state MEG activity, like all other activity in the body, is regulated between some “normal” range, and it is this range that manifests itself as the slight change in PLZC values between the limits observed. Furthermore, as the changes in the complexity with time all lay in a similar range with similar trends, the notion that this could be showing the nominal dynamics of the resting state complexity of MEG background activity is plausible. Thus, with the above in mind, due to the minimal variation of PLZC values, a lack of practical significance probably outweighs statistical significance of results. Therefore, complexity changes (calculated from the MEG time series) within this narrow range can be regarded as being stable. However, a limitation to this analysis that must be noted is that the minimal variation in PLZC may have become diluted in the remaining analyses performed as the PLZC values were averaged over the 5 min using the PLZC values for each 1 min epoch.

### 4.2. Effects of Age on Complexity Values

Similar to other analyses in literature [2,16,31], age has a significant effect on characteristics of MEG signals; in this particular case the complexity values. In this study, peak mean complexity values were observed in group 2 (mid-adulthood, 41–60years), which corresponds with the age when the brain has reached full maturity [31]. This result is similar to those from analyses done by Fernandez et al. [4] using LZC and Shumbayawonda et al. [16] using PE, where an increase in complexity and entropy, respectively, was observed after the brain reached full maturity. Although ageing is generalised over 3 age groups, the results show that female anterior complexity decreases more between groups 2 and 3 as compared to that of males. Thus, this implies that female complexity values are affected by age more than those of males [4].

Variations in the evolution of PLZC values for males and females with age can be attributed to a range of factors including: changing hormonal levels, the environment, employment, education, maintenance of resting default networks, synaptic pruning, changes in energy consumption, changes in axonal volumes, as well as the effects of maturation and ageing on the brain [30,31,48,49,50]. Thus, it is possible that the combined effects of these factors can have a direct/indirect effect on background MEG signals, which in turn will have a direct effect on the complexity values, resulting in the changes observed throughout this study.

### 4.3. Effects of Gender on Complexity Values

Gender analyses showed that, in general, males had higher complexity values than females in all 3 age groups, as shown in Figure 5. Results from this analysis showed that, apart from the left lateral (groups 1) and anterior region (group 2), PLZC values between males and females were significantly different. However, it is interesting to note that despite the existence of significant gender differences, analysis results (results shown in Figure 1) showed that the range of whole head PLZC values for both genders overlapped, with very small differences between males and females. The significance and impact of these gender differences are difficult to ascertain as they could be as a result of a combination of aspects including the effects of changes in various brain structures (such as white matter and grey matter changes [30]) between genders, the effects of hormones, differing education levels, the environment, or even some other unknown factors [1,3,30,51].

### 4.4. Interaction Effects of Age and Gender on Complexity Values

When the significance of the interaction between age and gender on whole head PLZC values was evaluated, it was found that this interaction was significant only in groups 1 and 3. Further investigations using 3-way ANOVA to identify the interaction effect of these two aspects in each brain region showed that the interaction effect was significant over the anterior region (groups 1 and 3), central region (all groups), left lateral region (groups 2 and 3), posterior region (all groups), and right lateral region (all groups). These results are similar to those observed by [4] where they observed a significant interaction effect in the anterior region of the brain. However, unlike the results obtained using LZC in [4], males were found to have higher PLZC values than females. Nevertheless, similar to analyses performed using LZC, females had a more prominent decline in PLZC values (from MA to LA) when compared to males in the same age range.

### 4.5. Potential Clinical Singificance of Results

In this study an investigation into the effects of age, gender, and the stability of complexity—calculated with PLZC—of the resting state in MEG recordings from healthy participants was made. The work carried out here would contribute to a fingerprint characterising healthy ageing, obtained by applying signal processing to different recordings capturing brain function (MEG in this particular case). Pathology can cause changes to brain activity that could alter the normal pattern of evolution of brain function with healthy ageing. Therefore, such fingerprint describing healthy ageing would allow comparing results from future studies and determining whether they fall under the healthy ageing category or show abnormal characteristics that might reflect some underlying pathological brain conditions.

### 4.6. Limitations and Future Work

The first limitation to this study was that the human lifespan was generalised from multiple subjects in this cross-sectional study; the effects of age may be more suitably addressed using longitudinal studies. However, as the computation of PLZC was done for each individual before averaging, it is our view that the results obtained from this comprehensive study can still be used to understand the changes in complexity with ageing. Secondly, the number of male and female subjects in each age group was not the same; this could have introduced a bias in the results. Future studies with the same number of males and females in each group are needed to confirm our preliminary results. Third, our study covered frequencies between 1.5 Hz and 40 Hz, and thus covered the entire spectrum of brain oscillations at rest. Although it is well known that the dominant waves during rest are the alpha waves, in this study, the complexity changes in this frequency band were not investigated as there is strong evidence that broadband activity is required for a proper evaluation of complexity in recordings of electromagnetic brain activity [52]. Nevertheless, future analysis to investigate complexity changes in the alpha band could be carried out to evaluate its role in healthy ageing. Lastly, as it is possible that source space analysis can yield more information about the sources of the brain regions which resulted in the changes observed in this sensor space study, source space analysis using PLZC should be done.

## 5. Conclusions

It was our aim to identify changes in complexity (PLZC) of the background MEG signals due to recording time, as well as the effects of age and gender on complexity values across the human lifespan. This study evaluated the changes in the complexity values of resting state MEG signals from healthy participants with ages ranging between 21 and 80 years. Investigation into the input parameters to be used in the analysis resulted in the identification of the optimal motif length to analyse MEG recordings using PLZC. PLZC values changed during the recording, but this change was small, in absolute terms, and suggested that complexity remained relatively stable during the 5 min of MEG recordings. Moreover, when age effects were analysed, it was found that for both genders, similar to other studies in literature, complexity values increase with age until mid-adulthood after which they began to decrease. However, female anterior complexity decreased more after mid-adulthood as compared to that of males which implied that female complexity values were affected by age more than those of males. Lastly, this study managed to highlight that males generally had higher average complexity values than females throughout life, with this difference being most clear in late-adulthood. The results from our study can be used to form a fingerprint of brain activity (recorded in MEGs) representing different stages of healthy ageing forming a baseline for detecting deviations from the “norm”, This would allow researchers and clinicians to compare their MEG results with the fingerprint and, potentially, assist with the diagnosis of different conditions.

## Figures and Tables

**Table 1:** Grouping of subjects according to age, with further classification of age groups into different stages of life that fall under maturation and ageing of the brain as described by Ge et al. [30] and Peters [31].

Group	Age	Subjects			Category
		Male	Female	Total	
1	21–40	34	35	69	Early Adulthood (EA)
2	41–60	21	16	37	Mid adulthood (MA)
3	61–80	24	47	71	Late adulthood (LA)

**Table 2:** Results from the pairwise comparisons of results obtained using different motif lengths (*m* = 3, 5, and 7). Results show that there are high correlations between complexity values obtained using motif lengths of 5 and 7. The highest significant correlations have been highlighted using bold font.

Comparisons		*m* = 3 and *m* = 5	*m* = 5 and *m* = 7	*m* = 3 and *m* = 7
Group 1	M	Correlation = 0.615	Correlation = **0.906**	Correlation = 0.417
		*p* < 0.0001	*p* < 0.0001	*p* < 0.0001
	F	Correlation = 0.381	Correlation = **0.907**	Correlation = 0.118
		*p* < 0.0001	*p* < 0.0001	*p* = 0.152
Group 2	M	Correlation = 0.354	Correlation = **0.768**	Correlation = −0.108
		*p* < 0.0001	*p* < 0.0001	*p* = 0.191
	F	Correlation = 0.577	Correlation = **0.940**	Correlation = 0.451
		*p* < 0.0001	*p* < 0.0001	*p* < 0.0001
Group 3	M	Correlation = 0.354	Correlation = **0.768**	Correlation = −0.108
		*p* < 0.0001	*p* < 0.0001	*p* = 0.191
	F	Correlation = 0.507	Correlation = **0.762**	Correlation = 0.193
		*p* < 0.0001	*p* < 0.0001	*p* = 0.019
